# Phase IIb Trial for the Palliative Treatment of Patients With Primary Hepatic Malignancy Unable to Receive Curative Treatment: Efficacy of Colchicine

**DOI:** 10.1002/kjm2.70121

**Published:** 2025-09-27

**Authors:** Zu‐Yau Lin, Ming‐Lun Yeh, Po‐Cheng Liang, Shinn‐Cherng Chen, Chung‐Feng Huang, Jee‐Fu Huang, Chia‐Yen Dai, Ming‐Lung Yu, Wan‐Long Chuang

**Affiliations:** ^1^ Division of Hepatobiliary Medicine, Department of Internal Medicine Kaohsiung Medical University Hospital Kaohsiung Taiwan; ^2^ Department of Internal Medicine, Faculty of Medicine, College of Medicine Kaohsiung Medical University Kaohsiung Taiwan; ^3^ Department of Internal Medicine, Faculty of Post‐Baccalaureate Medicine, College of Medicine Kaohsiung Medical University Kaohsiung Taiwan; ^4^ Center for Cancer Research Kaohsiung Medical University Kaohsiung Taiwan; ^5^ Center for Liquid Biopsy and Cohort Research Kaohsiung Medical University Kaohsiung Taiwan; ^6^ Department of Internal Medicine Kaohsiung Medical University Gangshan Hospital Kaohsiung Taiwan

**Keywords:** colchicine, hepatocellular carcinoma, immunotherapy, intrahepatic cholangiocarcinoma, tyrosine kinase inhibitor

## Abstract

This trial was to evaluate the efficacy and the safety of colchicine for the palliative treatment of patients with primary hepatic malignancy unable to receive curative treatment. Forty hepatocellular carcinoma (HCC) patients and two intrahepatic cholangiocarcinoma (ICC) patients signed the informed consents. Most HCC participants (97%) had failed in tyrosine kinase inhibitor (TKI) and/or immunotherapy before entering the study. Colchicine was started from 1 mg twice per day and was adjusted ranging from 1.5 to 3 mg/day. One treatment cycle was defined as four continuous treatment days followed by 3 days off. The HCC control group was matched to the same condition (Child score, tumor staging, previous TKI, and/or immunotherapy) as the participants at a ratio of 3 to 1 (control to colchicine). The ICC control group was matched to the same tumor staging as the participants. The primary objective was to compare the survival between the two groups. The safety objective was to observe the adverse events of colchicine. The colchicine HCC group demonstrated longer median survival (283 days) than the control group (107 days) (*p* < 0.0001, 95% confidence interval 2.001–3.289, hazard ratio 0.3513, 95% confidence interval 0.2611–0.5523). Two ICC participants survived 491 and 461 days, respectively, compared to the control group with a median survival of 8.5 months and a 41.5% one‐year survival rate. Diarrhea (5%) was the only directly colchicine‐related grade 3–4 adverse event. In conclusion, this colchicine dosage schedule is clinically feasible as an effective palliative treatment for patients with primary hepatic malignancy unable to receive curative treatment.

**Trial Registration:** ClinicalTrials.gov identifier: NCT04264260.

## 1 Introduction

The most frequent primary liver cancer in adults is hepatocellular carcinoma (HCC), comprising 75%–85% of cases, followed by intrahepatic cholangiocarcinoma (ICC), comprising 10%–15% of cases [1]. Curable managements include operative resection, local ablation, and liver transplantation, which are the first choice in the treatment of these patients. Patients unable to receive curable management can receive transcatheter arterial chemoembolization (TACE) or radioembolization, hepatic arterial infusion chemotherapy, radiation therapy, immunotherapies, or targeted therapy for the purpose of either downstaging for further curative treatment or prolongation of survival. Immunotherapy and targeted therapy with tyrosine kinase inhibitors (TKI) are two approved modalities to treat unresectable HCC. Although the overall positive response rate for immunotherapy is better than TKI, the efficiencies of immunotherapy alone or combined with TKI are still not satisfactory [2, 3]. On the other hand, the positive response rates for the treatment of ICC unable to receive curative management are only modest, even with immunotherapies or targeted therapy [4]. Moreover, targeted therapy and immunotherapy are expensive and may have non‐negligible side effects. Therefore, searching for a new drug to compensate for the insufficient efficiency of current treatments for unresectable hepatic malignancy is of clinical importance.

Colchicine is a very cheap lipophilic tricyclic alkaloid which has been used in medicine for a very long time [5, 6, 7, 8]. Colchicine at high concentration has a strong binding capacity to tubulin to perturb the assembly dynamics of microtubules [6, 9, 10]. It also can increase cellular free tubulin to limit mitochondrial metabolism in cancer cells through inhibition of the voltage‐dependent anion channels of the mitochondrial membrane [11]. However, the clinical application of colchicine as an anti‐cancer agent has not been accepted due to its toxicity and very narrow therapeutic range [6, 10, 12, 13, 14]. However, careful adjudgment of oral colchicine dosage schedule and exclusion of contraindications can minimize its possible side effects [5, 6, 14]. A clinically feasible colchicine dosage schedule had been shown to have the potential for the palliative treatment of advanced HCC in a phase IIa clinical trial [15]. This phase IIb open‐label non‐randomized trial was to evaluate the efficacy and the safety of this novel colchicine dosage schedule for the palliative treatment of patients with primary hepatic malignancy unable to receive curative treatment. Since the goal for colchicine treatment is not to obliterate malignancy, tumor progression with time is an expected result. In clinical practice, the duration of survival and the quality of life are the main concerns for the patients. Therefore, median survival and the safety issue rather than time to tumor progression (TTP) and tumor progression‐free survival (PFS) are the main concerns for this trial.

## 2 Patients and Method

From January 30, 2020, to December 31, 2023, a total of 42 participants (40 HCC, two ICC) collected from our institution signed the informed consents. The treatment and follow‐up period were ended on May 30, 2025. The American Joint Committee on Cancer (AJCC) TNM staging system 8th edition was applied for tumor staging. This study was approved by the institutional review board of the hospital, and all patients signed the informed consents. All procedures were performed in compliance with relevant laws and institutional guidelines.

### 2.1 Selection Criteria

#### 2.1.1 Inclusive Criteria

Patients with primary hepatic malignant tumors were unable to receive curative treatment. This indicated that the malignancy could not be eliminated by operative resection, liver transplantation, or local ablation therapy. Patients also needed to fit at least one of the following two criteria: (1) evidence of distant metastasis or large vessels (intrahepatic first branch portal vein, portal main trunk, major hepatic vein, or inferior vena cava) invasion, (2) failure in controlling malignancy by transcatheter arterial chemoembolization evidenced by the rapid appearance of new rather than incompletely treated nodules within 3 months after chemoembolization or technical failure in chemoembolization. The performance status of the patient based on the Eastern Cooperative Oncology Group (ECOG) belonged to 0 or 1, and the calculated Child score was below 8 points.

#### 2.1.2 Exclusive Criteria

These included (1) life‐threatening hemorrhage, (2) life‐threatening bacterial, fungal, or viral infection (excluding hepatitis B and/or C virus), (3) extrahepatic original malignancy unable to be controlled, (4) serum creatinine level > 1.5 mg/dL, (5) receiving long‐term statin or fibrates unable to be reduced in dose, (6) white blood cell count < 1500/μL, platelet count < 30,000/μL, or hemoglobin < 9.0 g/dL after medication, (7) under or plans for pregnancy, (8) allergy to colchicine or history of severe side effects caused by colchicine, (9) systemic chemotherapy within 2 months before enrollment or plans for receiving systemic chemotherapy, (10) under or plans for receiving other clinical trial medication, Chinese traditional medicine, or herbal drugs, (11) consumption of other clinical trial testing drugs within 3 months before enrollment, (12) severe malfunction of vital organs justified by the member of the research team, (13) under or plans for hospice care, (14) drug abuser or continued alcohol drinking > 30 g/day.

### 2.2 Dosage Schedule

The dosage schedule for colchicine was according to a previous study [15]. Colchicine (0.5 mg/tablet) was started at two tablets after meals twice per day and was adjusted ranging from the minimum 1.5 mg/day to the maximum 3 mg/day based on the tolerance of the participant. One treatment cycle was defined as four continuous treatment days followed by 3 days off. The treatment cycles were continuously repeated but could be temporarily discontinued based on the condition of the participants.

### 2.3 Adjustment of Colchicine Dosage

1. The daily colchicine dose was reduced 0.5 mg in participants with Child score 8–9 points, was returned to the original dose after Child score < 8 points, and was stopped in participants with Child score > 9 points.
2. Colchicine was temporarily stopped in participant suffering severe diarrhea (> 3 times of watery or not‐formed stool passages per day over baseline). The treatment cycle was started again with a reduced total daily dose after improvement of the symptom.
3. Colchicine was temporarily stopped when the participant fitted any one of the exclusive criteria during the study. The treatment cycle was started again after the fitted exclusive criterion was eliminated.
4. Colchicine was temporarily stopped one day before TACE until the participant showed no fever, the same hepatic reserved function as before, and a serum creatinine level < 1.5 mg/dL after embolization.
5. Colchicine was temporarily reduced to half of the original dose when the participant temporarily received a P‐Glycoprotein inhibitor or a strong Cytochrome P450 3A4 inhibitor.

### 2.4 Withdrawal Criteria

1. Participant suffered from severe or untolerable side effects of colchicine such as systemic itching, diarrhea, nausea, vomiting, abdominal pain, fever, or skin rash.
2. Participant was unable to tolerate the total daily dose of 1.5 mg colchicine for more than 4 cycles. This does not include temporarily reducing the dose to a total of 1 mg during the temporary application of a P‐Glycoprotein inhibitor or a strong Cytochrome P450 3A4 inhibitor.

### 2.5 Concomitant Treatment

1. The following treatments were permitted during the study, including non‐curative operative resection of the tumor, local ablation therapy, local radiation therapy, transcatheter arterial chemoembolization, target therapy, and immunotherapy.
2. The following treatments were prohibited, including systemic chemotherapy, other clinical trial testing drugs, Chinese traditional medicine, and herb drugs.

### 2.6 Follow‐Up Procedures

Ultrasonography, contrasted‐enhanced computed tomography, or magnetic resonance imaging was performed within every 3 to 4 months. Chest X‐ray and whole‐body bone scan were performed based on the condition of the participants. Serum tumor markers such as alpha‐fetoprotein or CA19‐9 (carbohydrate antigen 19–9) were determined at least one session within 3 months. The complete blood count, hepatic, and renal function were determined at least one session every month. The participants were asked to visit investigators' outpatient clinic at least one session every month.

### 2.7 Selection of Control Group

#### 2.7.1 HCC

The control group was originated from patients (From January 30, 2020, to December 31, 2023) treated by members of this research team matched the same condition (Child score, tumor staging, previous TKI and/or immunotherapy) as the participants at the time of entering the study (three patients in the control group to one participant in the colchicine group).

#### 2.7.2 ICC

The control group was originated from the collection of patients (From January 2019 to December 2023) treated in our hospital, matching the same tumor staging as the participants at the time of entering the study.

### 2.8 Objectives

The primary objective was to compare the median survival between participants receiving colchicine for more than 4 cycles and the control group. The safety objective was to observe the adverse events of colchicine. The adverse events were recorded based on the Common Terminology Criteria for Adverse Events (CTCAE) Version 5.0 published on November 27, 2017.

### 2.9 Statistical Analysis

The Fisher exact test or chi‐squared test was applied to compare proportions between two groups. The Mann–Whitney U test was applied to compare the medians of two groups. Survival analysis was calculated by the log–rank test, and the median survival, 95% confidence interval (CI), and the hazard ratio were applied to evaluate the colchicine efficacy. The safety assessment is presented in a narrative statistical manner for the collection of adverse events.

## 3 Results

Seven HCC participants early withdrew (≤ 4 cycles) from the study and were excluded. The reasons for withdrawal were rapid exacerbation of hepatic function due to tumor progression in two patients, side effects of colchicine including nausea and vomiting in one patient, and self‐withdrawal due to no confidence in colchicine in the remaining four patients. Overall, 33 HCC participants and two ICC participants were included for analysis. Almost all participants in the HCC group (97%) had experience of failure in TKI and/or immunotherapy before entering the study (Table 1). Eleven HCC participants also received combined TKI and/or immunotherapy during the study. The other 14 HCC participants did not receive combined TKI and/or immunotherapy due to side effects of drugs and/or economic considerations (Table 2). There was no significant difference in either previous TKI and/or immunotherapy or concomitant therapy between the HCC colchicine group and the control group.

**TABLE 1:** Characteristics of participants.

	Hepatocellular carcinoma (*n* = 33)	Intrahepatic cholangiocarcinoma (*n* = 2)
Age (years)	40–74, 64	38, 64
Sex (male/female)	28/5	0/2
Etiology (HBV/HCV/alcohol/unknown)	21/7/1/4	2/0/0/0
Liver cirrhosis (+/−)	28/5	0/2
TNM staging^a^		
Hepatocellular carcinoma		
II	4	
IIIB	13	
IVB	16	
Intrahepatic cholangiocarcinoma		
IV		2
Previous target therapy and/or immunotherapy		
Sorafenib	1	
Sorafenib + regorafenib	14	
Lenvatinib	8	
Sorafenib + lenvatinib	1	
Sorafenib + lenvatinib + immunotherapy	2	
Sorafenib + regorafenib + lenvatinib	5	
Lenvatinib + Immunotherapy	1	
None	1	2
Total colchicine treatment cycles	6–147, 24	51, 59
Reasons for discontinuation of colchicine^b^		
Tumor progression	22	2
Exacerbation of liver function	5	
Systemic chemotherapy	1	
Self‐withdrawal	3	
Death not related to cancer	1	

*Note*: Data in hepatocellular carcinoma group was described as range and median.

Abbreviations: HBV, hepatitis B virus; HCV, hepatitis C virus.

^a^ The tumor stage was determined on enrollment based on the American Joint Committee on Cancer TNM staging system 8th edition.

^b^ One hepatocellular carcinoma participant is still receiving colchicine after the end of the follow‐up date.

**TABLE 2:** Comparison the characteristics between HCC colchicine group and control group.

	Colchicine group (*n* = 33)	Control group (*n* = 99)	*p*
(a) Age(years)	40–74, 64	41–86, 66	0.0394
(b) Sex (male/female)	28/5	66/33	0.0457^a^
(c) Liver cirrhosis (+/−)	28/5	87/12	0.7645
(d) Etiologies (HBV/HCV/alcohol/unknown)	21/7/1/4	56/27/1/15	0.7078
(e) Previous TKI or immunotherapy			
HCC Staging^b^			
II	1 (L), 1 (S + *R*), 1 (S + L), 1 (S + L + I)	2 (S), 1 (L), 6 (S + *R*), 1 (S + L), 1 (S + L + I), 1 (S + *R* + L + I)	
IIIB	5 (L), 7 (S + *R*), 1 (S + L + I)	1 (I), 18 (L), 6 (S), 7 (S + *R*), 4 (L + I), 1 (S + I), 2 (S + *R* + I)	
IVB	1 (none), 1 (S), 2 (L), 6 (S + *R*), 1 (L + I), 5 (S + *R* + L)	3 (none), 4 (S), 11 (L), 8 (S + *R*), 3 (S + L), 3 (*R* + L), 5 (L + I), 1 (S + I), 3 (S + *R* + L), 3 (L + *R* + I), 2 (S + *R* + I), 2 (S + *R* + L + I)	
Total	1 (none), 29 (TKI), 3 (TKI + I)	3 (none), 73 (TKI), 23 (TKI + I)	0.2067
(f) Concomitant treatment			
HCC Staging			
II	1 (none),1 (TACE + RT), 1 (TACE + RT + I), 1 (TACE + L + I)	4 (none), 3 (TACE), 1 (RT), 1 (I), 2 (TACE + I), 1 (TACE + RFA + I)	
IIIB	4 (none), 4 (TACE), 1 (RT), 1 (TACE + I), 1 (TACE + L), 1 (TACE + RFA + I), 1 (TACE + RFA + L)	19 (none), 2 (L), 2 (I), 6 (RT), 1 (TACE), 1 (L + I), 1 (RT + I), 1 (RT + S), 1 (RT + L), 1 (RFA + RT), 2 (TACE + RT), 1 (RT + TACE + I), 1 (S + I + TACE + RT)	
IVB	7 (none), 1 (RT), 1 (L), 1 (S), 1 (TACE + RFA), 1 (TACE + RT), 1 (RT + I), 1 (TACE + I), 1 (TACE + RT + RFA), 1 (TACE + RT + L)	27 (none), 3 (I), 2 (L), 1 (S), 4 (RT), 1 (TACE), 2 (L + I), 1 (*R* + I), 1 (RT + L), 1 (TACE + I), 2 (TACE + RT), 1 (S + *R* + I), 1 (RT + *R* + L), 1 (TACE + *R*T + S + R + I)	
Total	12 (none), 10 (local therapy^c^), 2 (TKI and/or I), 9 (local therapy + TKI and/or I)	50 (none), 21 (local therapy), 16 (TKI and/or I), 12 (local therapy + TKI and/or I)	0.0616

*Note*: Data were described as range and median.

Abbreviations: HBV, hepatitis B virus; HCC, hepatocellular carcinoma; HCV, hepatitis C virus; I, immunotherapy (nivolumab, pembrolizumab, or atezolizumab plus bevacizumab); L, lenvatinib; R, regorafenib; RFA, radiofrequency ablation; RT, radiation therapy; S, sorafenib; TACE, transcatheter arterial chemoembolization; TKI, tyrosine kinase inhibitor.

^a^ The chi‐square statistic with Yates correction was 3.1534. The *p*‐value was 0.0758 and was not significant.

^b^ The tumor stage was determined on enrollment based on the American Joint Committee on Cancer TNM staging system 8th edition.

^c^ Local therapy included TACE, RT, and RFA. The Mann–Whitney U test, chi‐square test, and Fisher exact test were applied for analysis.

### 3.1 Survival

#### 3.1.1 HCC

**FIGURE 1:**
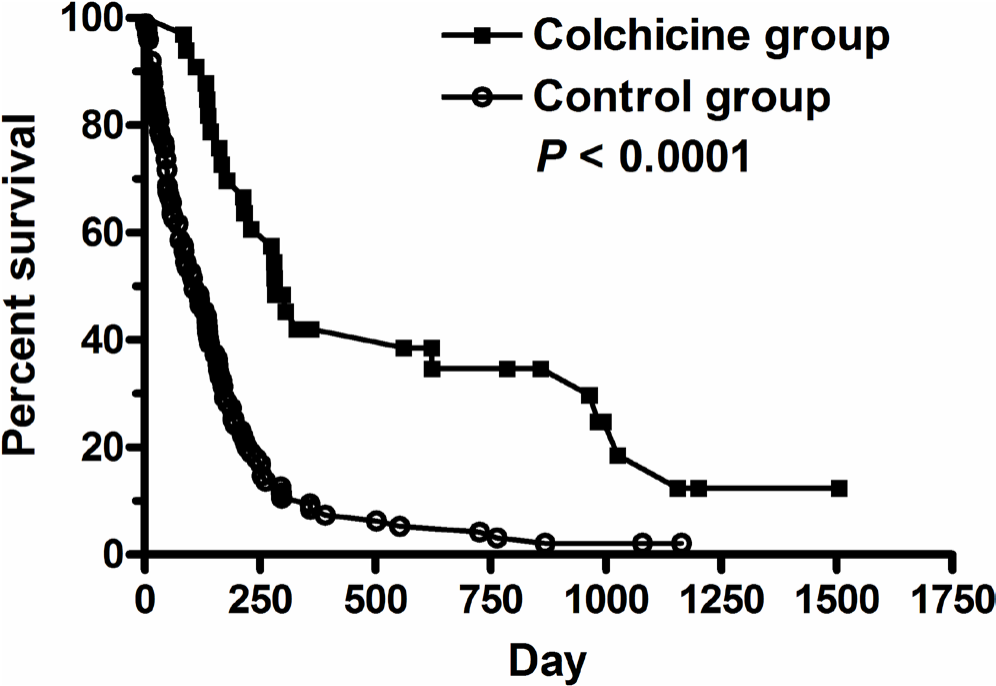
Comparison the survival between colchicine group (*n* = 33) and control group (*n* = 99) in hepatocellular carcinoma patients. The log–rank test was applied for statistical analysis. *p* < 0.0001.

Comparison of the characteristics between the colchicine group and the control group was shown in Table 2. The control group was slightly older than the colchicine group. Although the control group showed more female patients than the colchicine group, calculated by the chi‐square test, there was no significant difference after Yates correction. There was no significant difference in the incidence of previous TKI or immunotherapy or concomitant therapy between the colchicine group and the control group. The colchicine group showed longer median survival (283 days) than the control group (107 days) (*p* < 0.0001, 95% CI of ratio 2.001–3.289, hazard ratio 0.3513, 95% CI of ratio 0.2611–0.5523) (Figure 1).

#### 3.1.2 ICC

The first 38 years old participant showed intrahepatic tumor (> 5 cm) with multiple bone metastases and metastatic lymph nodes in the hepatoduodenal ligament and left paraaortic region. The second 64 years old participant showed intrahepatic tumor (> 5 cm) with right subphrenic, right pleural, and right lower lung invasion, metastases at right lower lung, and thrombus in the inferior vena cava and right antrum. Both participants had not been treated by systemic chemotherapy, target therapy, or immunotherapy, and the second participant had been treated by TACE and radiation therapy before entering the study. The first participant received a combination of colchicine, TACE, and radiation therapy, and the second participant received a combination of colchicine and radiation therapy. The first participant survived for 491 days, and the second participant survived for 461 days. The control group included 56 patients, with a median survival of 8.5 months, and the one‐year survival rate was 41.5%.

### 3.2 Adverse Events

For grade 1–2 adverse events observed in the colchicine group (Table 3), diarrhea was the most common event (75%). The other grade 1–2 adverse events with an incidence of > 10% of participants included abdominal pain (37.5%), anorexia (25%), upper gastrointestinal hemorrhage (20%), an increase in serum creatinine level (12.5%), an increase in bilirubin level (12.5%), and pruritus (12.5%). For grade 3–4 severe adverse events in the colchicine group (Table 4), anemia was the most common event (20%). The other grade 3–4 severe adverse events with an incidence of ≥ 5% of participants included an increase in serum bilirubin level (12.5%), body weight loss (7.5%), diarrhea (5%), a decrease in platelet count (5%), dyspnea (5%), and biliary tract infection (5%).

**TABLE 3:** Grade 1 or 2 adverse events in colchicine group.

Event	Number of episodes	Involved participants (%)^a^
Diarrhea	64	30 (75%)
Abdominal pain	23	15 (37.5%)
Anorexia	15	10 (25%)
Upper gastrointestinal hemorrhage	16	8 (20%)
Serum creatinine increased	14	5 (12.5%)
Pruritus	9	5 (12.5%)
Blood bilirubin increased	6	5 (12.5%)
Nausea/vomiting	6	4 (10%)
Herpes zoster	5	4 (10%)
Edema limbs	5	3 (7.5%)
Proteinuria	5	3 (7.5%)
Dizziness	3	3 (7.5%)
Fatigue	2	2 (5%)
Dyspnea	2	2 (5%)
Lung infection	2	2 (5%)
Arthritis	1	1 (2.5%)
Hair loss	1	1 (2.5%)
Hoarseness	1	1 (2.5%)
Lower gastrointestinal hemorrhage	1	1 (2.5%)
Hypoglycemia	1	1 (2.5%)
Dysgeusia	1	1 (2.5%)
Hyponatremia	1	1 (2.5%)
Epistaxis	1	1 (2.5%)
Ascites	1	1 (2.5%)
Biliary tract infection	1	1 (2.5%)

*Note*: The adverse events were recorded based on the Common Terminology Criteria for Adverse Events (CTCAE) Version 5.0 published on November 27, 2017.

^a^ Two early withdrawal participants without taking colchicine were excluded from the calculation.

**TABLE 4:** Severe adverse events in colchicine group.

Event	Number of episodes	Involved participants (%)^a^
Anemia	14^b^	8 (20%)
Blood bilirubin increased	5	5 (12.5%)
Weight loss	3	3 (7.5%)
Diarrhea	4	2 (5%)
Platelet count decreased	4	2 (5%)
Dyspnea	2	2 (5%)
Biliary tract infection	2	2 (5%)
Lung infection	1	1 (2.5%)
Intracranial hemorrhage	1	1 (2.5%)
Abdominal infection	1	1 (2.5%)
Edema limbs	1	1 (2.5%)
Serum creatinine increased	1	1 (2.5%)
Hyponatremia	1	1 (2.5%)

*Note*: The definition of severe adverse event (grade ≥ 3) was based on the Common Terminology Criteria for Adverse Events (CTCAE) Version 5.0 published on November 27, 2017.

^a^ Two early withdrawal participants without taking colchicine were excluded from calculation.

^b^ The reasons for anemia were caused by tumor rupture in four episodes, gastric varices rupture in one episode, and peptic ulcer in nine episodes.

## 4 Discussion

Colchicine by oral intake is rapidly absorbed by the small intestine with a significant serum level within 1 h of administration [16]. This drug is mainly metabolized by the liver, with 80% of its excretion via the gastrointestinal tract [16], and liver cirrhosis can slow down its metabolism [17]. The estimated half‐life ranges from 26 to 31 h [16, 18, 19, 20]. The peak plasma concentrations after oral administration of 0.6–1 mg clinically acceptable colchicine dose range from approximately 2–6 ng/mL [18, 19, 20]. Since most patients with HCC also combine chronic hepatitis and/or cirrhosis, long‐term persistent administration of high colchicine doses can easily induce severe toxicity due to accumulation. Fortunately, previous IIa clinical trial using a novel dosage schedule had shown its clinical potential to overcome colchicine limitations in these patients [15]. Therefore, we conducted this IIb trial to further evaluate the efficiency and safety of this novel dosage schedule.

Previous in vitro and in vivo experiments had shown that 2–6 ng/mL colchicine concentrations had dose‐dependent anti‐cancer effects on HCC cells [21], cancer‐associated fibroblasts [21], and cholangiocarcinoma cells [22]. Both direct colchicine‐tubulin interaction [8, 9, 10, 11] and colchicine‐induced differential expressions of anti‐proliferative genes [21, 22] are two explanations for the anti‐cancer effects of colchicine on both HCC and cholangiocarcinoma cells. Moreover, previous in vitro studies also demonstrated that combined clinically achievable plasma colchicine concentrations with sorafenib or lenvatinib could promote the total anti‐cancer effects on HCC [23, 24], and colchicine could be added to regorafenib non‐responders [23]. Therefore, the HCC colchicine group showed significantly longer median survival than the control group, and two ICC participants also demonstrated better survival time than the control group.

Diarrhea, which was the most common grade 1–2 adverse event, is the most well‐known side effect of colchicine treatment. The symptom of diarrhea could usually could be prevented or controlled by reducing the colchicine dose and/or adding drugs such as dioctahedral smectite or loperamide on colchicine treatment days. For the other grade 1–2 adverse events, only anorexia and pruritus were considered to be colchicine‐related. These symptoms could be obliterated by temporary discontinuation of colchicine. Colchicine could be tried again with a reduced dose. The other grade 1–2 adverse events had no strong relation to colchicine administration and were symptomatically treated. Although anemia was the most common grade 3–4 severe adverse event observed in the colchicine group, the causes were not considered to be directly colchicine‐related. Tumor progression, pre‐existing liver cirrhosis causing rupture of varices, and peptic ulcer were the causes. Grade 3–4 adverse events of increased serum bilirubin level and body weight loss were considered to be caused by tumor progression. The other grade 3–4 adverse events, except for diarrhea, were also considered not to be colchicine‐related. On the other hand, the cost of colchicine administration based on this novel dosage schedule is much cheaper than either targeted therapy or immunotherapy. This allows us to have a long‐term prescription of this drug without causing any economic burden.

The present study was unable to select a fixed anti‐cancer drug in the HCC control group for comparison due to the complexity of previous TKI and/or immunotherapy in participants. To overcome this potential limitation of the study design, patients in the HCC control group were carefully matched to the same condition as the participants at the time of entering the study. Moreover, there was no significant difference in concomitant therapy between the HCC colchicine group and the control group. The present study thus still can offer valuable insights into the efficacy and safety of colchicine in the treatment of these patients. The complexity of previous TKI and/or immunotherapy in participants can be explained by the fact that these managements are proven standard systemic therapies for HCC. Since the efficacy of colchicine in HCC is still under investigation, patients will certainly be treated with TKI and/or immunotherapy at first due to ethical considerations. Only patients who failed or were unable to receive these standard managements were included in the study. Based on the results of this study design, the efficacy of colchicine was regarded as palliative management. On the other hand, TTP and PFS are frequently applied in developing a new drug in oncology. An advantage of measuring TTP or PFS over measuring survival is that TTP or PFS takes a much shorter duration of trial than measuring survival. However, PFS or TTP improvement does not always result in corresponding improvement in survival [25, 26, 27]. Therefore, median survival was applied to evaluate the potential efficiency of colchicine in HCC.

In conclusion, this colchicine dosage schedule is clinically feasible as an effective palliative treatment for patients with primary hepatic malignancy unable to receive curative treatment. This conclusion warrants further investigation in a definitive phase III trial.

## Consent

This study was approved by the institutional review board of the hospital (KMUHIRB‐F(II)‐20,190,152), and all patients signed the informed consents.

## Conflicts of Interest

The authors declare no conflicts of interest.

## Data Availability

Data sharing not applicable to this article as no datasets were generated or analysed during the current study.
